# A Rational Approach to Predicting Immediate Release Formulation Behavior in Multiple Gastric Motility Patterns: A Combination of a Biorelevant Apparatus, Design of Experiments, and Machine Learning †

**DOI:** 10.3390/pharmaceutics15082056

**Published:** 2023-07-31

**Authors:** Marcela Staniszewska, Michał Romański, Sebastian Polak, Grzegorz Garbacz, Justyna Dobosz, Daria Myslitska, Svitlana Romanova, Jadwiga Paszkowska, Dorota Danielak

**Affiliations:** 1 Physiolution Polska, 74 Piłsudskiego St., 50-020 Wrocław, Poland; g.garbacz@physiolution.pl (G.G.); j.dobosz@physiolution.pl (J.D.); d.myslitska@physiolution.pl (D.M.); s.romanova@physiolution.pl (S.R.); j.paszkowska@physiolution.pl (J.P.); 2 Department of Physical Pharmacy and Pharmacokinetics, Poznan University of Medical Sciences, 3 Rokietnicka St., 60-806 Poznań, Poland; michalroman@ump.edu.pl (M.R.); danielak@ump.edu.pl (D.D.); 3 Faculty of Pharmacy, Medical College, Jagiellonian University, Medyczna 9 Street, 30-688 Kraków, Poland; sebastian.polak@uj.edu.pl

**Keywords:** biopredictive dissolution testing, individual dissolution profiles, design of experiments, machine learning, motility patterns, mechanical stress events

## Abstract

Gastric mechanical stress often impacts drug dissolution from solid oral dosage forms, but in vitro experiments cannot recreate the substantial variability of gastric motility in a reasonable time. This study, for the first time, combines a novel dissolution apparatus with the design of experiments (DoE) and machine learning (ML) to overcome this obstacle. The workflow involves the testing of soft gelatin capsules in a set of fasted-state biorelevant dissolution experiments created with DoE. The dissolution results are used by an ML algorithm to build the classification model of the capsule’s opening in response to intragastric stress (IS) within the physiological space of timing and magnitude. Next, a random forest algorithm is used to model the further drug dissolution. The predictive power of the two ML models is verified with independent dissolution tests, and they outperform a polynomial-based DoE model. Moreover, the developed tool reasonably simulates over 50 dissolution profiles under varying IS conditions. Hence, we prove that our method can be utilized for the simulation of dissolution profiles related to the multiplicity of individual gastric motility patterns. In perspective, the developed workflow can improve virtual bioequivalence trials and the patient-centric development of immediate-release oral dosage forms.

## 1. Introduction

The modeling of formulations’ performance considering the variability of physiological conditions represents a novel and indispensable aspect of the biopredictive testing of oral medicines. It comprises the simulation of the predominant factors that influence dosage form disintegration and drug dissolution. Besides the composition of gastrointestinal (GI) liquids and fluctuations in physico-chemical parameters, such as pH and temperature, these include the often forgotten timing and magnitude of mechanical stress events [1,2,3,4]. The mechanical agitation occurring in the human stomach under fasting conditions creates a pattern known as a gastric interdigestive migrating motor complex, but the individual pressure waves—their strength and timing—are variable and difficult to predict. Research by Schneider et al. on physiological conditions in a fasted state showed that in over half of the examined subjects, the intragastric pressure events of magnitude between 100 and approximately 450 mbar were recorded, but all nine subjects experienced multiple minor pressure events [5]. The authors described the pressure activity of a stomach as highly variable, since patients differed with the timing, pressure, and number of stress events. Numerous studies have shown that the mechanical properties of formulations are among the critical factors that should be considered in biorelevant dissolution tests simulating either fasting or postprandial conditions [6,7,8,9,10,11]. Such an example is the research by Garbacz et al. on the release of diclofenac sodium from extended-release (ER) tablets [12]. The goal of their study was to elucidate the irregular peaks in the plasma concentration profiles in human plasma after the fasted administration of the ER tablets. It was hypothesized that the sensitivity of a formulation to mechanical stress was responsible for the variable absorption; thus, the authors tested dosage forms in an in-house-designed biorelevant apparatus—a stress test device—that simulated GI passage [13]. The biorelevant conditions were reflected in the rotation of the basket with the dosage form and sequences of pressure waves applied. The in vitro test results demonstrated the significant impact of the pressure events on the subsequent release of the drug from the tablets and the insensitivity of pellets toward such stresses. The following simulations of the plasma profiles confirmed the hypothesis. In the aforementioned study, the authors proposed three test protocols, intended to simulate rapid, regular, and late complete gastric emptying (GE). This allowed the estimation of the formulations’ properties under the boundary and mean conditions of the simulated GI tract. The approach of testing a dosage form under protocols taking into account extreme conditions in a gastric compartment has been adopted by other authors, also for immediate-release (IR) tablets and capsules [14,15]. On the other hand, none of these test programs has focused on the variability of intragastric stress (IS) events but rather on the timing of the culminating “housekeeper wave”. Hence, not only one test protocol but a set of protocols is needed to reflect the human gastric motility patterns of clinical study subjects [9,16], since their variability is large [5,17,18]. The well-established biorelevant methods—such as TIM-1 [19] or DGM [20]—approach the formulation testing assuming fixed test protocols, reflecting averaged groups of patients, rather than generating multiple individual dissolution profiles of the simulated clinical study population. Such individual dissolution profiles could become valuable input for the simulation of individual pharmacokinetic (PK) profiles and further virtual clinical trials. The following question arises: is there a method to better understand the formulation performance and susceptibility to mechanical agitation with as few dissolution tests as possible? The design of experiments (DoE) is a group of statistical methods commonly known for balancing the resources (number of experiments to perform) with the knowledge gained [21]. DoE has been applied to a broad range of engineering problems [22], but also plays a role in pharmaceutical development: finding the composition of a formulation of predefined properties, developing an optimal analytical method, or optimizing the process parameters of a dosage form’s production [23]. Some of the DoE approaches, under the common name of the response surface methodology (RSM), describe the effect of each tested variable on the process’ response with a polynomial regression model [24]. This feature has been employed to build empirical models of the dissolved amount of a drug in time as a function of formulation variables (concentrations of excipients) [25,26,27] or to optimize the conditions of a standard or bi-phasic dissolution test (paddle speed, organic volume, and pH gradient) [23,28]. To the authors’ knowledge, the application of the DoE methods to design in vitro drug dissolution tests simulating gastric motility has not been reported to date. Not only a rational method for the design of dissolution test scenarios in a physiological range of factors is needed, but also a workflow for the subsequent modeling and simulation of the dissolution profiles obtained from such tests. This seems to be of the highest importance for dosage forms that are characterized by disintegration due to IS events, including tablets and hard or soft capsules [29]. For capsules, one strategy is the mechanistic modeling of their disintegration under biorelevant conditions. Supposedly, the magnitude of pressure events and dissolution of the capsules’ shells (including the shell’s composition and thickness, the temperature of the dissolution medium, its composition, and the time of immersion) should be taken into account, but, to the authors’ knowledge, the scientific literature lacks such a sophisticated model at the moment. Another path is constructing an empirical model that describes the dissolved amount of the drug as the total effect of all the possible phenomena, such as the capsule’s disintegration, drug dissolution, and—if it occurs—precipitation. Such a model can be generated using supervised machine learning (ML) tools. They operate via an iterative procedure involving the exposition of a learning dataset to the algorithm that constructs relationships between input and output vectors [30]. Among these methods, there are classification and regression algorithms: classification models serve to model discrete values (classes), and regression models operate with continuous outputs. The most prominent example of the utilization of ML algorithms in pharmaceutical research is drug discovery [31], but these tools have also been employed for the design of drug formulations [32] and the modeling of in vitro–in vivo relationships [33]. The aim of this study was to develop a method for the prediction of the drug release from an IR dosage form—a soft gelatin capsule—in the two-dimensional space of the fasted-state-relevant timing and magnitude of the intragastric pressure wave. First, we used DoE to efficiently distribute the test conditions in the design space. Then, we modeled the dissolution profiles using a logistic regression algorithm and a random forest algorithm. We hypothesized that coupling DoE with biorelevant dissolution testing and ML algorithms could establish a novel and efficient workflow to achieve the study goal. In the future, the developed workflow can be used to understand better the performance of oral dosage forms under in vivo conditions.

## 2. Materials and Methods

### 2.1. Materials

Ketokaps Max soft gelatin capsules with a solution of ketoprofen free acid 50 mg (PPF Hasco-Lek S.A., Wrocław, Poland) were purchased from a local pharmacy. Ketoprofen of pharmaceutical grade was used as a working standard. Sodium chloride 99.9%, potassium dihydrogen phosphate (Reag. Ph. Eur, 99.7%), sodium hydroxide pellets (Reag. Ph. Eur, 99.1%), acetonitrile (HPLC gradient grade), and orthophosphoric acid 85% were purchased from VWR Chemicals (Leuven, Belgium); hydrochloric acid 20% was purchased from Chemsolute (Renningen, Germany).

The composition of the simulated gastric fluid (SGF) included NaCl (2 g/L) dissolved in deionized water and acidified to pH 1.8 with 20% HCl. For the simulated intestinal fluid (SIF, Biorelevant, London, UK) concentrate, KH_2_PO_4_ (68 g/L) was dissolved in deionized water, and the pH of the solution was adjusted to 7.4 with 40% *w*/*w* NaOH.

### 2.2. Dissolution in PhysioCell

#### 2.2.1. Apparatus

The dissolution experiments were performed with PhysioCell—a novel multicompartmental dissolution apparatus for the simulation of the upper gastrointestinal tract [34]. The key features of the apparatus are the ability to simulate pressure events of up to 500 mbar, mimicking the gradients of the dissolution medium flow rate and temperature, the rapid neutralization of the gastric content, and dynamic pH control and adjustment with hydrogen carbonate buffers. These were proven to be crucial for biorelevant dissolution tests [35,36,37,38,39]. PhysioCell consists of three vessels: the StressCell, Overflow Vessel, and Collection Vessel. In this study, PhysioCell was operating in a closed-loop mode with only the StressCell connected with a dissolution medium reservoir. PhysioCell recently proved its usefulness for biorelevant dissolution tests of IR tablets, serving as a basis for in vitro–in vivo predictions (IVIVP) [40].

#### 2.2.2. Dissolution Test Conditions

In total, 900 mL of the SGF was provided in temperature and flow rate gradients. The temperature gradient included an increase in temperature from room temperature to 37 ℃ in approximately 15 min, and the flow rate gradient was introduced with 1-min intervals as follows: 50.0, 20.0, 17.0, 15.0, 13.5, 12.3, 11.3, 10.5, 9.9, 9.4, 9.0, 8.7, 8.4, 8.2, and 8.0 mL/min, until the end of the test. Moreover, gentle contractions of the elastic sleeve were utilized with a frequency of 1 per minute to enhance the mixing of the content of the StressCell without disturbing the integrity of the capsule. The capsule was placed in an in-house-designed elastic sinker (figure available in the Supplementary Materials) that prevented its floating and ensured the fixed localization of the dosage form in the StressCell [41]. In all test programs, the biorelevant pressure waves were introduced for the simulation of the IS events and GE. The programs involved the different timing (between 8 and 16 min) and strength (between 80 and 220 mbar) of the single intragastric pressure wave (see Section 2.4). The GE was always simulated at 30 min of the test and consisted of a sequence of three pressure waves of 300 mbar and was followed by an elevated flow rate. Moreover, during the GE, 100 mL of the SIF concentrate was introduced to the dissolution medium reservoir for the neutralization of SGF and simulation of the intestinal conditions. The final pH of the dissolution medium was 6.8. Sampling took place at predefined time points: 3 and 6 min, then every minute from 9 to 30 min, and every 3 min up to 60 min. The samples of 1 mL were manually withdrawn from the dissolution medium reservoir through a polyethylene cannula filter and filtered through a syringe filter (regenerated cellulose, 0.22 µm) into a vial. Ketoprofen was determined in the samples with a validated HPLC-UV method, described in the Supplementary Materials.

### 2.3. Software

The matrix of dissolution experiments was designed and DoE was executed in the Develve Statistical software (v. 4.14.1.0, Develve, Velp, Netherlands) [42]. The calculations of the logistic regression and random forest algorithms were performed with the Waikato Environment for Knowledge Analysis (Weka, v. 3.8.6, Hamilton, New Zealand) [43,44]. The data were visualized with the R statistical software (v. 4.1.2, R Core Team 2021, Vienna, Austria).

### 2.4. Matrix of Dissolution Tests

The central composite design (CCD) was employed to form a matrix of dissolution experiments with the stress events’ timing and pressure evenly distributed within physiological ranges (8–16 min and 80–220 mbar, respectively) (see Table 1)—these are later referred to as the basic tests. Moreover, four verification tests with intermediate values of pressure and timing were performed to evaluate the predictive power of the models.

### 2.5. Modeling and Evaluation of Dissolution Profiles

The initial approach was to utilize the DoE method to construct the model of the dissolution profiles as a function of the pressure wave’s timing and magnitude. For this model, only the results of 13 basic dissolution tests served as input data. Once the model was generated, the dissolution profiles for the four verification tests were calculated and compared with the actual results. Since it was found that the DoE modeling inaccurately described the dissolution from the capsules that did not start disintegrating in response to the simulated IS event, ML algorithms were used. The first step in this approach was the construction of a classification model that described whether a pressure event of given timing and magnitude initiated the capsule’s disintegration. Moreover, three more dissolution tests were performed to capture the formulation’s behavior under critical conditions when the capsule began to be susceptible to pressure events, which was between 9 and 12 min. These tests (see Table 1) are later referred to as the additional tests. Hence, not only the basic but also the additional test results were used as the input data to the ML classification algorithm to increase the precision of the model. The second step was the modeling of the dissolution profiles with a random forest algorithm. As the ketoprofen solution was released from the capsule to the pH 1.8 SGF until the GE at 30 min, and then to pH 6.8 SIF, the observed dissolution profiles were a result of the capsule’s disintegration, drug precipitation, and drug redissolution.

#### 2.5.1. DoE Model of Drug Dissolution

The CCD, belonging to the RSM, provided a mathematical model of the response—which, in this case, was defined as the percentage amount of a drug (*D*) dissolved at each sampling time point (*i*)—as a function of two factors: IS timing (*t*) and IS pressure (*p*). Thus, the model involved a series of polynomials with the coefficients (*b*) that were estimated in the Develve software, based on the results of the 13 basic tests:

(1) $$D_{i}=b_{i,0}+b_{i,1}p+b_{i,2}t+b_{i,12}pt+b_{i,11}p^{2}+b_{i,22}t^{2}$$

Moreover, the Develve software estimated the *p*-value of each coefficient, calculated with ANOVA, and the authors assumed the coefficients of *p*-values of less than 0.05 to be statistically significant.

#### 2.5.2. Classification Model: Logistic Regression Algorithm

The model classified the capsules into “open”—those that started disintegrating during the pressure event—and “closed”—those that remained intact until the simulated GE, despite the IS event. The input data for this model consisted of the basic and additional tests, and each test was manually assigned to the *open* or *closed* class labels. Hence, the independent variables in the input data were the IS timing and pressure, and the response was open/closed class. The following default parameters were applied for the calculations performed in the Weka Environment: *weka.classifiers.functions.Logistic-R 1.0E-8-M-1-num-decimal-places 4*. The software also returned the confidence for the prediction (z), which accounted for the estimated probability of the predicted open/closed class.

#### 2.5.3. Regression Model: Random Forest Algorithm

The second step in the ML approach utilized the random forest algorithm for the modeling of the dissolution profiles. The input data consisted of the predicted open/closed class from the first step, the timing and pressure of the IS events, and the dissolution profiles from the basic and additional tests. The algorithm operated with the following setup: *weka.classifiers.trees.RandomForest-P 100-I 5000-num-slots 1-K 1-M 1.0-V 0.001-S 1-batch-size 2 and with 10-fold cross-validation*.

#### 2.5.4. Evaluation of the Predicted Dissolution Profiles

The evaluation of the two models was based on a visual assessment of the alignment of the predicted dissolution profiles with the experimental ones. Moreover, we analyzed the predicted profiles that were obtained for the dense values of the timing and pressure of the IS event, other than the experimental ones. The key investigated features were the models’ capability to predict the capsule’s disintegration start after the IS time and the minimal conditions of IS timing and pressure magnitude triggering the disintegration. The predictive power of the models was verified with the four independent dissolution tests from the verification set. The goodness of fit of the predicted dissolution profiles to the actual ones was assessed by means of visual examination, mean absolute error (MAE), and root mean square error (RMSE). The visual examination relied on the point-by-point comparison of the profile shapes and the number of the predicted points falling outside the uncertainty region, resulting from the properties of the capsule and apparatus. The variability of the capsule’s performance was established using the standard deviation (SD) in the dissolved drug amounts observed at the sampling times. It was calculated from the results obtained in the central point dissolution tests no. 1, 4, 7, 10, 13. The dissolution profiles were divided into three time sections: before the IS event, between the IS and the GE, and after the GE. The maximal SD value was determined for each section. Accounting for the inaccuracy of the medium flow rate in the PhysioCell, provided by the peristaltic pump, the dissolved drug amount observed at a given sampling time (D_obs,i_) was associated with the uncertainty interval from D_obs,i−1_ to D_obs,i+1_, denoting the actual dissolved amounts at the previous and next sampling times, respectively. Eventually, the model predictions in the verification tests were expected to fall within the acceptance interval from D_obs,i−1_− 2 SD to D_obs,i+1_ + 2 SD.

#### 2.5.5. Simulating Dissolution Profiles for Customizable IS Parameters

The primary goal of the study was to develop a methodology that enabled the reliable prediction of the dissolution profiles for a variety of IS timing and magnitude pairs, particularly those that had not been tested experimentally. For this purpose, the two models were utilized for the calculation of the profiles for the new multiple combinations of the IS parameters: with regard to the impact of pressure (simulated stresses at 9, 9.5, 10, 10.5, 11, 12, 13, 14, 15 min; pressure range: 100–200 mbar) and the impact of timing (simulated magnitude of 100, 160, and 200 mbar; timing range: 9–15 min).

## 3. Results

### 3.1. Dissolution Results

**Figure 1:**
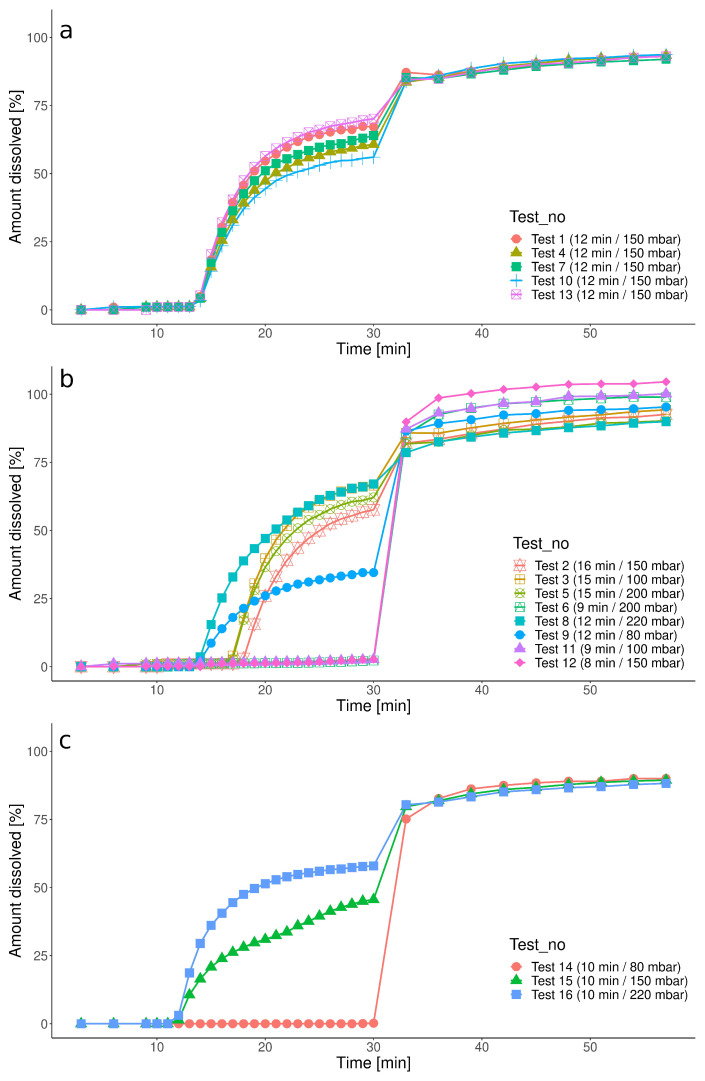
Dissolution results of Ketokaps obtained under biorelevant conditions in the PhysioCell: (**a**) experiments in the center point, (**b**) experiments in the factorial and star points, (**c**) additional tests.

Figure 1 presents the experimental dissolution results obtained in the basic and additional tests described in Table 1. The scheduled stress events significantly impacted the capsules’ disintegration start times and further drug dissolution. The experiments in the center point simulated the IS event of 150 mbar applied at 12 min, and it triggered the disintegration, which was associated with the rapid dissolution (Figure 1a). These experiments also assessed the repeatability of the dissolution process since they were performed under the same nominal conditions. These five dissolution profiles were characterized by moderate variability, with a maximum value of the relative standard deviation of 8.8% at 29 min. The experiments in the factorial and star points (Figure 1b) showed two opposite reactions of the capsules towards IS events: the capsules squeezed at early time points (Tests 6, 11, 12) were resistant towards the pressure wave, regardless of the pressure magnitude. Later stress timing with either lower or greater magnitude initiated the disintegration. Figure 1c presents additional tests with fixed timing of the IS event (10 min) and varying magnitude. Only the pressure wave magnitude equal to or greater than 150 mbar opened the capsule, and the magnitude of this effect correlated with the pressure applied. Such a phenomenon was also observed in the basic test set (test 9 vs. test 8). Additionally, we observed increased medium opacity after the pressure event that could indicate ketoprofen precipitation. This effect could be attributed to the limited volume of the StressCell (ca. 25 mL), despite the total medium volume of 900 mL circulating in the whole system before the GE, which was sufficient to provide sink conditions [45]. The simulated GE caused a sudden burst in drug dissolution, because all the remaining drug was evacuated from the capsule and was subsequently dissolved by a large portion of the dissolution medium of the elevated pH flowing through the StressCell. Therefore, complete dissolution was obtained. Of note, the sampling site location caused an approximately 2-min delay between the nominal time of the pressure wave and the timing of the increase in the dissolved drug amount in the recorded dissolution profile.

### 3.2. The Predictions of the Verification Test Set

**Figure 2:**
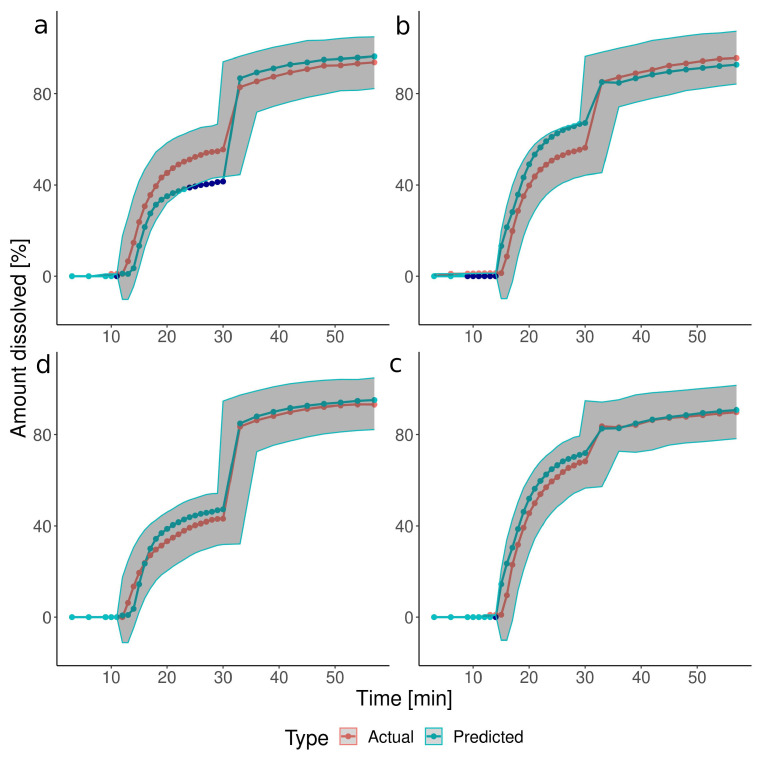
The comparison of the experimental (orange) with the CCD model-predicted (light blue) dissolution profiles in the verification tests: (**a**) Test V1, IS at 10.5 min, 175 mbar; (**b**) Test V2, IS at 13.5 min, 175 mbar, (**c**) Test V3, IS at 13.5 min, 125 mbar, (**d**) Test V4, IS at 10.5 min, 125 mbar. Dark blue points depict the predicted data points falling outside the acceptance intervals (see Section 2.5.4) shown as dark-grey ribbons.

**Figure 3:**
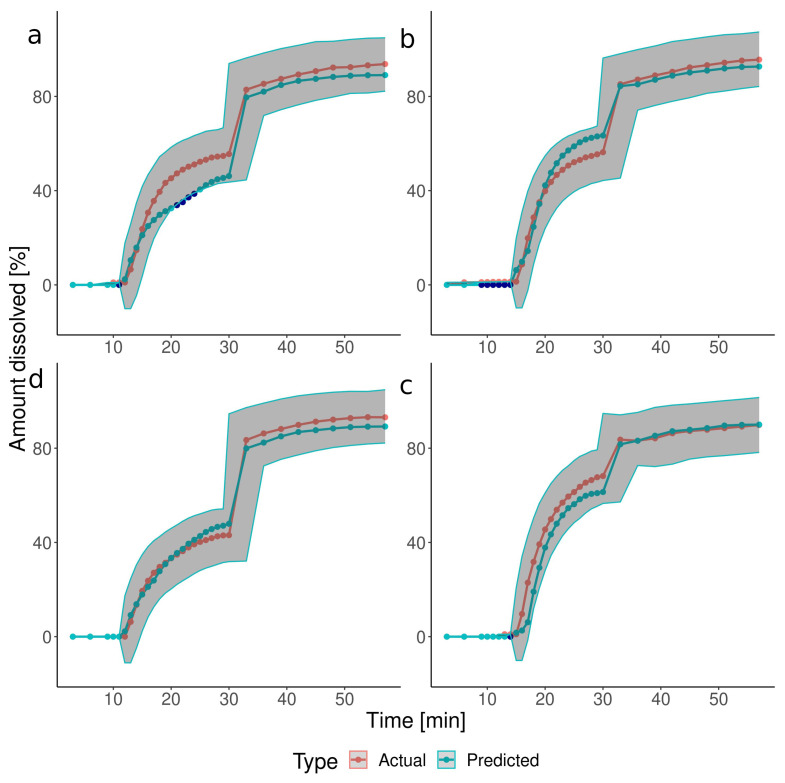
The comparison of the experimental (orange) with the ML model-predicted (light blue) dissolution profiles in the verification tests: (**a**) Test V1, IS at 10.5 min, 175 mbar; (**b**) Test V2, IS at 13.5 min, 175 mbar, (**c**) Test V3, IS at 13.5 min, 125 mbar, (**d**) Test V4, IS at 10.5 min, 125 mbar. Dark blue points indicate the predicted data points falling outside the acceptance intervals (see Section 2.5.4) shown as dark-grey ribbons.

The CCD described the dissolution profiles with a series of polynomials. The coefficients of the polynomials and their *p*-values are available in the Supplementary Materials, together with the postdictions of the basic and additional tests. These equations were utilized to simulate the dissolution in the four independent verification experiments (Figure 2). The predicted dissolution profiles mostly matched the actual values, with 11% of points falling outside of the acceptance interval. However, certain discrepancies in the prediction of the disintegration start time occurred: a 1–2-min delay in the predicted values for tests V1 and V4 and too early opening for tests V2 and V3. Statistical analysis of the CCD parameters revealed that mostly the IS timing, timing*timing, and constant terms of the polynomials were statistically significant in predicting the dissolved drug amounts at time points between 19 and 30 min. It indicated that the timing of the IS, not the pressure, predominantly impacted the shape of the dissolution profiles in this time range. Similarly to CCD, the ML-predicted dissolution profiles in the verification tests resembled the experimental ones, and only 9% of points fell outside the defined acceptance interval (Figure 3). Moreover, the ML method better approximated the onset of the capsule’s disintegration, with only a small delay of 1 min for test V2. It resulted in an improvement in the goodness-of-fit parameters (MAE: 3.793 vs. 4.605, RMSE: 5.245 vs. 6.258 for ML and CCD, respectively).

### 3.3. Simulation of Dissolution under Customizable IS Parameters

#### 3.3.1. Impact of the IS Stress Magnitude

**Figure 4:**
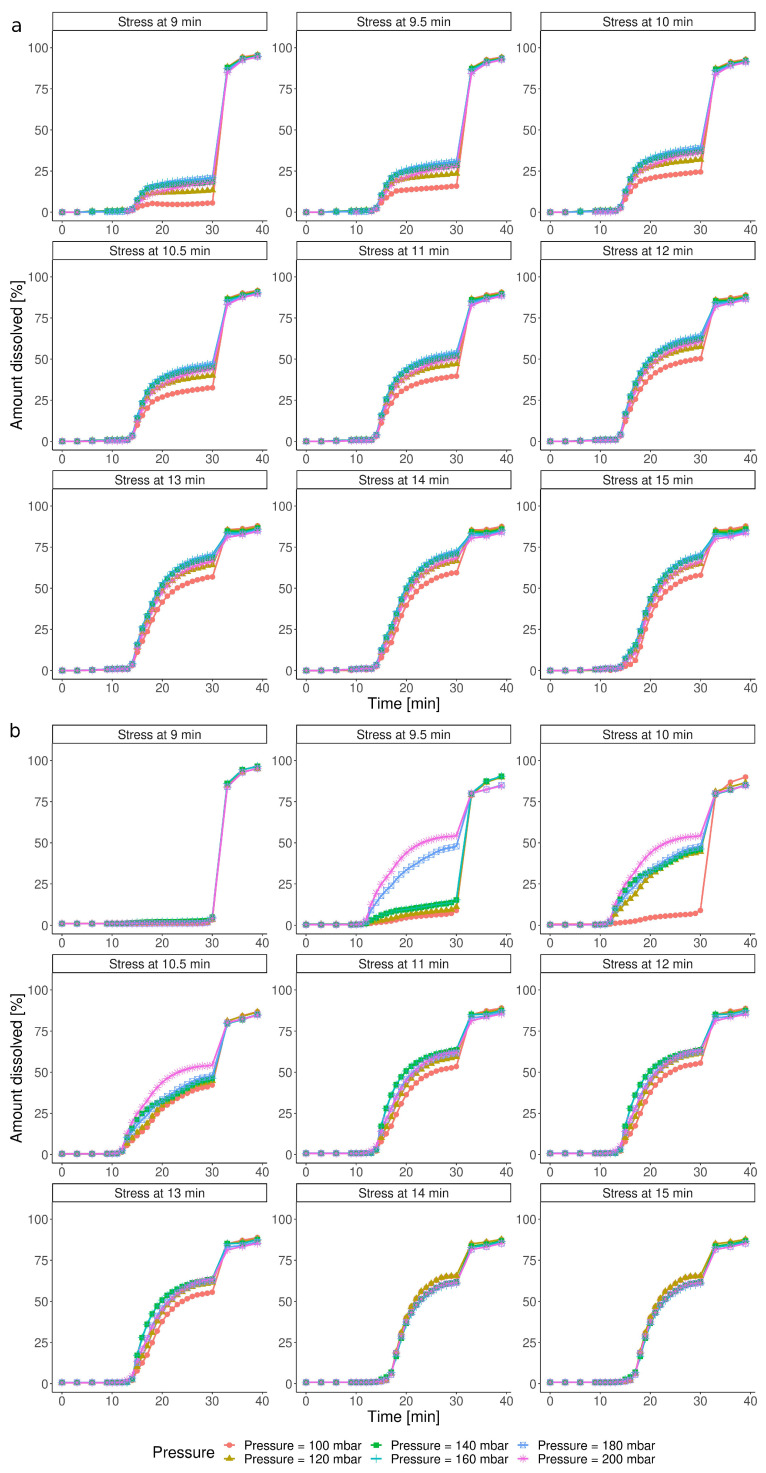
Impact of the pressure of the IS event at the predefined time points on the dissolution profiles simulated with (**a**) CCD and (**b**) ML.

Figure 4a presents simulations for the various combinations of the IS event parameters based on the CCD model. The simulation indicated that even the slightest IS, between 9 and 12 min, should break the capsule’s shell and initiate the dissolution, and this effect increased with the pressure magnitude. It contradicted the experimental results, where Ketokaps was resistant toward the stress events applied in 9 min (Figure 1b, test no. 6, 11, and 12). The obtained data suggested that the CCD model was not able to discriminate between open and closed cases. Such a model cannot be used to predict the minimal timing that triggers disintegration. The same cases simulated with ML were strikingly different (Figure 4b). According to this simulation, the Ketokaps were unaffected by the early stress event (9 min). If the pressure was applied only half a minute later, stress of 180 and 200 mbar could trigger disintegration, resulting in a profile characteristic for open capsules. The ML model predicted that if the IS was delayed further, the capsules would disintegrate more easily: with the stress at 10.5 min and later, each magnitude above 100 mbar would open a capsule. For IS timing over 11 min, the influence of the pressure magnitude was negligible since all the dissolution profiles were aligned.

#### 3.3.2. Impact of the Timing of the IS Stress

**Figure 5:**
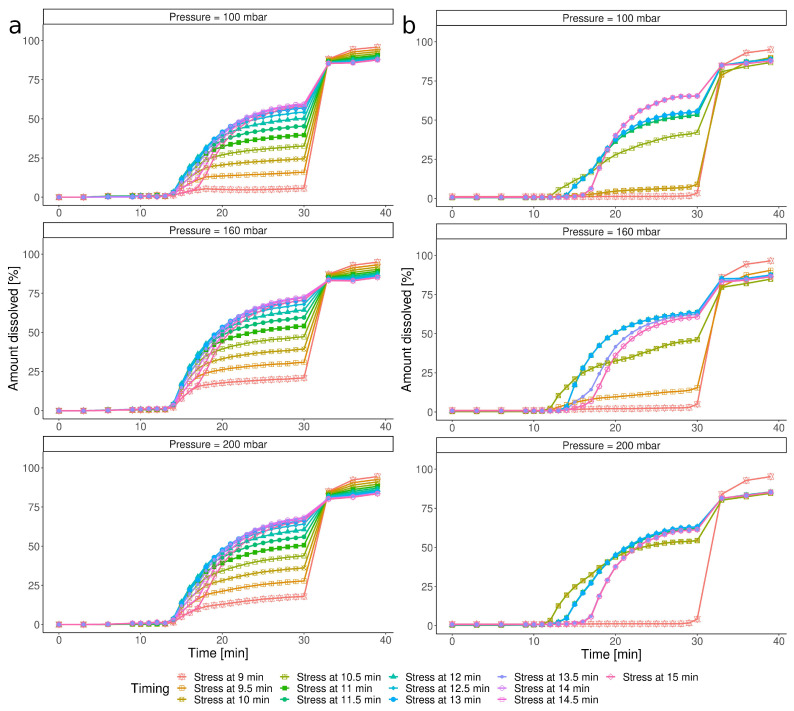
Impact of the timing of the IS event under constant pressure on the dissolution profiles simulated with (**a**) CCD and (**b**) ML.

Figure 5 shows how the two models described the impact of the timing of the stress event, given its constant pressure value. The CCD predicted that, in all cases, the capsules’ disintegration began at the same time point, regardless of the timing of the IS applied (Figure 5a). On the other hand, the ML approach not only distinguished among the *open/closed* states of the capsules, predicting that an early IS would not influence the dissolution, but also captured that the disintegration onset time may differ. In some profiles, IS caused a major increase in the amount dissolved (i.e., stress at 15 min), resulting in a dose-dumping-like event. In the others, the dissolution occurred gradually (i.e., stress at 10 min), which could be caused by the partial disruption of the capsule shell.

### 3.4. Susceptibility of the Capsule towards the IS Timing and Pressure

**Figure 6:**
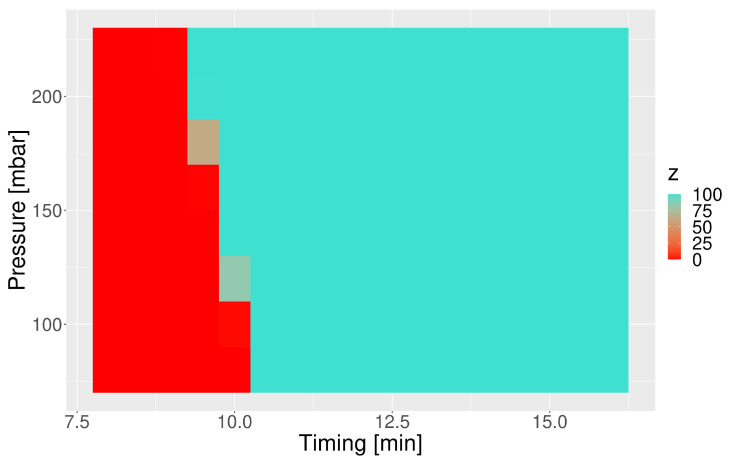
The confidence of the classification model’s assignment to the *open* (z = 100) or *closed* (z = 0) class.

The confidence of the predictions of the logistic regression model (z) was interpreted as an indicator of the sensitivity of capsules towards pressure waves, and it is graphically represented in Figure 6. The red tiles indicate conditions that would not trigger disintegration, while the turquoise ones indicate combinations of timing and pressure that would open the Ketokaps capsule. The predicted zones are aligned with the basic, additional, and verification test results. Most importantly, they expand the understanding of the formulation’s properties in the areas that have not been explored experimentally.

## 4. Discussion

In this study, we present a novel workflow for the simulation of a multiplicity of individual dissolution profiles of a model IR formulation—Ketokaps gelatin capsules—subjected to variable conditions characteristic of a fasting stomach. The conditions were reflected in the PhysioCell—a novel dissolution apparatus—and accounted for irregular gastric motility patterns via the application of a pressure wave of various magnitudes and timing. We used CCD to minimize the number of experiments, modeled the dissolution profiles with ML, and predicted the dissolution for the intermittent stress event conditions (timing and pressure) not used in the experiment. It allowed us to cover the space of the gastric motility patterns that could be relevant for pharmacokinetic simulations by means of 16 experiments only and, in turn, we obtained a large number of in silico dissolution profiles. Hence, the novel workflow is a time- and resource-efficient method to thoroughly investigate IR formulations’ performance under fasted-state simulated gastric conditions. This work is based on the optimal design of test scenarios in the PhysioCell device. We hypothesized that the timing of the stress event, its magnitude, and the interplay between them might be crucial for the disintegration of the soft gelatin capsules. This goal was achieved with the CCD applied to the factors, resulting in 13 experiments only, further supplemented with three more tests. If the standard approach of the full factorial design was used, as many as 25 experiments would have been necessary to check the impact of two factors at five levels. Thus, by application of the modified CCD, we evenly distributed the experiments in the test space and balanced the resources (number of tests), following the Quality by Design principles. In pharmaceutical research, 3, 6, and even 12 repetitions are routinely used to assess the variability at each experimental condition. The CCD estimates the system’s variability by replicating the center point only, thus reducing the number of the required test runs. We followed this approach to determine the variability of the experimental dissolution profiles.

An essential part of the research was the construction and verification of a model of drug dissolution as a function of the timing and the strength of the simulated IS event. This was achieved in two ways: with polynomial regression modeling based on CCD and by the implementation of the two-step ML approach. The CCD model demonstrated the importance of the stress event timing and predicted 89% of the points with acceptable accuracy. However, it was not successful in capturing the dynamics of the disintegration and dissolution processes; it predicted the dissolution profiles for the customizable pairs of the IS timing and magnitude as variants of the central point profiles, rather than treating them individually. ML and CCD/DoE are based on similar approaches, and DoE (understood as an umbrella rather than a specific model) can use exactly the same algorithms. The results of the current study suggest that algorithms more flexible than polynomials are needed to describe the phenomenon of interest. With this, if DoE utilized random forest to fit the data, the result should be the same as with the ML method. A successful case of the utilization of the DoE method to optimize biopredictive dissolution conditions and to build in vitro–in vivo correlations for an IR capsule was presented by Mercuri et al. [46]. Moreover, Carapeto et al. utilized DoE and physiologically based biopharmaceutics modeling to develop a biopredictive dissolution method [47]. However, these approaches applied multiple linear regression and analysis of variance to assess how the composition and volume of the dissolution media, apparatus type, dosage form, and rotational speed influenced the in vitro drug dissolution. In particular, they did not include the prediction of the individual dissolution profiles or the simulation of mechanical agitation on the dissolved drug amount. In our study, the impact of the IS timing and magnitude on the in vitro dissolution turned out to be too complex to model with a simple polynomial function. Moreover, Sun et al. pointed out that a polynomial model might oversimplify the dissolution process of a controlled-release system as a function of its composition [48]. Instead, the authors recommended the utilization of artificial neural networks. We also acknowledge the superiority of the ML learning algorithms over the polynomial model. Although the ML approach predicted the verification tests’ profiles with accuracy similar to the CCD model, it was superior in differentiating the conditions triggering the capsule’s disintegration and recognizing the correlation between the timing of the IS event and the capsule’s rupture. ML also provides more realistic predictions when the IS parameters fall outside the tested ranges; exemplary predictions are available in the Supplementary Materials. Our predictive ML model originated in the dissolution test scenarios methodically designed with the CCD, hence showing the complementarity, and not the exclusivity, of these methods, as discussed by Freiesleben et al. [49]. Although the present study was limited to a fixed dissolution medium pH and composition, GE timing, IS event number, and flow rate pattern, the workflow that we describe has the flexibility to model the impacts of other biorelevant parameters on drug dissolution. Their combinations would aid in complex simulations of various physiological conditions met in the fasted stomach, allowing a highly individualized approach to the evaluation of oral solid dosage forms. Such a study is under preparation and will be addressed in a separate paper. We also presume that the DoE and ML combination might be implemented in experiments with compendial apparatuses (USP 1-4) and conditions. Then, the input parameters would be, for example, the flow rate, paddle rotation, or glass bead size. It requires, however, confirmation in another study.

Another outcome of our work is the map of the susceptibility of the capsule toward pressure events (Figure 6). Such a map, prepared for a series of formulations, may serve to compare their mechanical properties and assess the risks of various times of disintegration onset. It is advantageous to standard texture analysis since it describes the formulation’s properties in a dynamic, two-dimensional matrix of conditions of the magnitude and timing of the pressure wave and estimates the response of the dosage form under conditions that were not tested experimentally. Moreover, the method could be beneficial in testing pressure-controlled formulations [32,50], such as 3D-printed capsules [51]. On the other hand, the current study was limited to the simulation of intensive pressure events of magnitude between 80 and 220 mbar, but the in vivo data show that the stomach wall displays also lower-pressure waves of 50 mbar or less [5]. An extension of the utilized magnitude range would produce even more biorelevant conditions. However, our analysis showed that it was the timing, not the strength of the pressure wave, that played a major role in the disintegration, at least for the Ketokaps. The workflow presented in this study focuses on the response of the IR formulation towards stress events. A potential application of the approach to extended-release drug products—for example, matrix tablets that often partially erode due to pressure events [52,53,54]—would presumably need some adjustments. Firstly, the use of the regression model alone would be more appropriate to describe drug dissolution. Secondly, multiple pressure events should be considered due to the high resistance of such formulations to mechanical agitation compared with IR products. Finally, it would be reasonable to use an apparatus dedicated to testing such dosage forms, such as the Advanced Modular Platform (Physiolution Polska), which is an upgraded version of the stress test [13]. However, these recommendations should be verified in a separate study and were not within the scope of this work.

The workflow presented in this paper allows the determination of the dissolution profiles not only under extreme or mean conditions of gastric motility but also in all customizable cases within these limits. Hence, we can simulate the formulation’s performance in various individuals with different gastrointestinal motility patterns. It would partially address the within- and between-subject variability already at the in vitro experimental level. Such a bottom-up approach could improve the IVIVP and further enable virtual bioequivalence (VBE) trials. A recent study by Bego et al. showed that the variability of certain GI tract parameters might be incorporated to simulate within-subject variability [55]. The authors also noticed that, due to the abundance of physiological factors that affect oral drug absorption, it would be impossible to evaluate them all experimentally, at least in the near future. Our workflow fills the gap in assessing formulation responses to the variable GI parameters and thus might assist in increasing the precision of VBE.

Another study by Loisios-Konstantinidis et al. explored the variability of physiological parameters in mechanistic physiologically based pharmacokinetic modeling for VBE [56]. The authors applied a variable GE time, stomach and intestinal pH, and bile salt concentration in the simulation of individual subjects for the VBE trials. The workflow included the first-order disintegration model; hence, it can be utilized for simple formulations only. However, biorelevant dissolution testing in apparatuses simulating mechanical agitation (e.g., PhysioCell) and including the gastric variability factors directly might be more beneficial.

The dissolution profiles simulated for individual patients might provide valuable input data for further pharmacokinetic simulations. The recent work of Mohylyuk et al. underlined the importance of the generation of individual dissolution profiles on the basis of separate GI scenarios [7]. Similarly to our study, the authors designed five dissolution test scenarios, but for an extended-release formulation, simulating various motility patterns with the application of 2 N mechanical loading in a texture analyzer on a tablet withdrawn after 1, 2, and 4 h from the dissolution medium in a USP2 apparatus. The obtained dissolution profiles were compared with deconvoluted plasma profiles for the establishment of an in vitro–in vivo correlation by weighting each test scenario. Although such an approach is reasonable, it is unclear if it could also predict the intermediate cases of GI motility since, at the moment, each test scenario describes one group of multiple patients. In contrast, the approach presented in our study might predict a formulation’s performance in a population of individual subjects—for example, in VBE. It is achieved using the parameters that prospectively characterize gastric motility within physiologically plausible ranges, instead of retrospectively analyzing clinical trials’ outcomes. Consequently, our approach enables a predictive, not retrospective, estimation of drug dissolution from the oral formulation. The clinical relevance of the method remains under investigation.

## 5. Conclusions

The present study describes a novel method for the prediction of formulation properties considering the variability of the fasted gastric conditions. The workflow includes the design of an experimental space through DoE and two-step modeling of the dissolution results with ML methods: logistic regression and random forest algorithms. These methods have been successfully applied to predict multiple individual dissolution profiles that might be utilized for further pharmacokinetic simulations.

## Figures and Tables

**Table 1:** Dissolution test conditions: timing and magnitude of the pressure wave.

Group	Test	Pressure [mbar]	Timing [min]
Basic tests	Test 1 *	150	12
	Test 2	150	16
	Test 3	100	15
	Test 4 *	150	12
	Test 5	200	15
	Test 6	200	9
	Test 7 *	150	12
	Test 8	220	12
	Test 9	80	12
	Test 10 *	150	12
	Test 11	100	9
	Test 12	150	8
	Test 13 *	150	12
Additional tests	Test 14	80	10
	Test 15	150	10
	Test 16	220	10
Verification tests	Test V1	175	10.5
	Test V2	175	13.5
	Test V3	125	13.5
	Test V4	125	10.5

* The center point that is located in the mathematical center of the variability range.

## Data Availability

Data are available on request.

## Supplementary Materials

The following supporting information can be downloaded: https://www.mdpi.com/article/10.3390/pharmaceutics15082056/s1, Table S1: HPLC_UV method conditions; Table S2: coefficients of polynomials with *p*-values; Figure S1: The elastic sinker; Figure S2: Exemplary predictions with IS parameters outside the tested ranges; Figure S3: Postdictions generated with the DoE model; Figure S4: Postdictions generated with the ML model.

## Abbreviations

The following abbreviations are used in this manuscript:

CCD	central composite design
DoE	design of experiments
ER	extended-release
GE	gastric emptying
GI	gastrointestinal
IR	immediate-release
IS	intragastric stress
IVIVP	in vitro–in vivo predictions
MAE	mean absolute error
MDPI	Multidisciplinary Digital Publishing Institute
ML	machine learning
PK	pharmacokinetic
RMSE	root mean square error
RSM	response surface methodology
SD	standard deviation
SGF	simulated gastric fluid
SIF	simulated intestinal fluid
VBE	virtual bioequivalence
