# Supplementary material for: A Rational Approach to Predicting Immediate Release Formulation Behavior in Multiple Gastric Motility Patterns: A Combination of a Biorelevant Apparatus, Design of Experiments, and Machine Learning
Source: Pharmaceutics. 2023 Jul 31;15(8):2056. doi: 10.3390/pharmaceutics15082056 (PMC10458881; doi:10.3390/pharmaceutics15082056)
Supplement: Supplementary file 1 [file pharmaceutics-15-02056-s001.zip › Table S1_HPLC-UV_method_conditions.pdf]

Column	ACE 3 C18 150 x 4.6 mm, 3 µm
Phase A	0.1% H_3PO_4 , pH 2.1
Phase B	ACN
Elution	Isocratic, A/B 45:55
Flowrate	1 mL/min
Column temperature	25 °C
Wavelength	255 nm
Injection volume	2 uL
Retention time	4.5 min
Linearity range	0.01 – 0.06 mg/mL

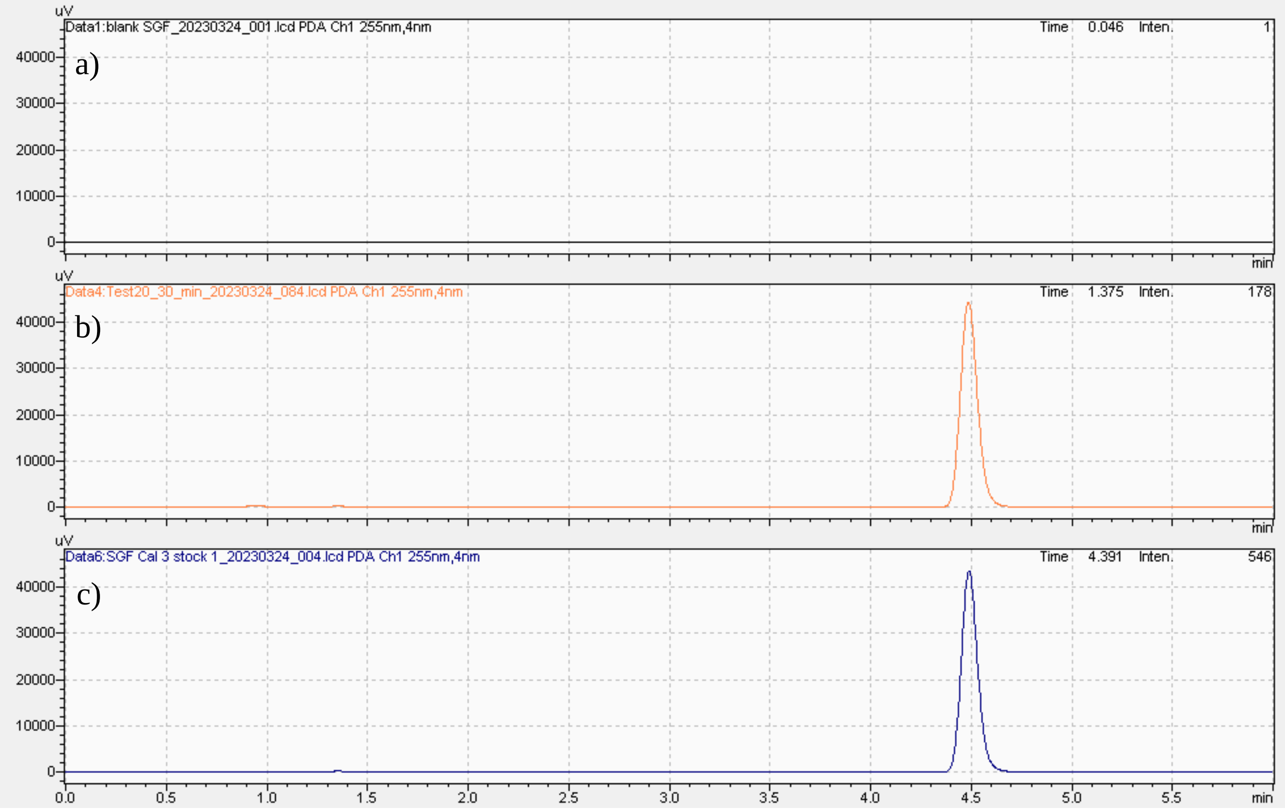

Figure 1. Exemplary chromatograms of a) blank SGF solution, b) dissolution test sample of ketoprofen in SGF and c) calibration solution of ketoprofen in SGF.
