# Supplementary material for: A Rational Approach to Predicting Immediate Release Formulation Behavior in Multiple Gastric Motility Patterns: A Combination of a Biorelevant Apparatus, Design of Experiments, and Machine Learning
Source: Pharmaceutics. 2023 Jul 31;15(8):2056. doi: 10.3390/pharmaceutics15082056 (PMC10458881; doi:10.3390/pharmaceutics15082056)
Supplement: Supplementary file 1 [file pharmaceutics-15-02056-s001.zip › Table S2_polynomial_coefficients.pdf]

		C	p	t	pt	p2	t2
D0	Coefficient	0.000	0.000	0.000	0.000	0.000	0.000
D3	Coefficient	0.000	0.000	0.000	0.000	0.000	0.000
D6	Coefficient	0.406	-0.134	-0.134	0.268	-0.136	-0.136
	p-value	0.105	0.464	0.464	0.309	0.486	0.486
D9	Coefficient	0.866	-0.016	-0.016	0.523	-0.302	-0.302
	p-value	0.004	0.924	0.924	0.059	0.129	0.129
D10	Coefficient	1.106	-0.145	0.108	0.300	-0.352	-0.352
	p-value	0.001	0.417	0.543	0.247	0.092	0.092
D11	Coefficient	1.128	-0.151	0.288	0.318	-0.420	-0.163
	p-value	0.000	0.276	0.059	0.123	0.018	0.275
D12	Coefficient	1.150	-0.033	0.157	0.750	-0.362	-0.104
	p-value	0.002	0.863	0.415	0.778	0.105	0.608
D13	Coefficient	1.156	-0.031	0.162	0.073	-0.357	-0.094
	p-value	0.002	0.872	0.416	0.792	0.119	0.653
D14	Coefficient	4.408	0.194	0.165	0.078	-0.873	-2.080
	p-value	0.000	0.479	0.544	0.839	0.016	0.000
D15	Coefficient	17.242	1.170	-0.023	0.080	-3.943	-9.430
	p-value	0.000	0.393	0.986	0.966	0.024	0.000
D16	Coefficient	28.020	1.961	-0.030	0.088	-6.496	-15.749
	p-value	0.000	0.383	0.989	0.977	0.024	0.000
D17	Coefficient	36.086	2.432	0.533	-0.210	-8.026	-20.216
	p-value	0.000	0.374	0.841	0.955	0.022	0.000
D18	Coefficient	42.436	2.916	4.398	-0.153	-7.798	-21.836
	p-value	0.000	0.288	0.126	0.967	0.024	0.000
D19	Coefficient	47.214	3.045	9.517	-0.528	-8.171	-20.846
	p-value	0.000	0.304	0.010	0.896	0.027	0.000
D20	Coefficient	50.762	3.264	13.545	-0.715	-8.427	-19.914
	p-value	0.000	0.301	0.002	0.868	0.031	0.000
D21	Coefficient	53.592	3.438	16.384	-0.973	-8.516	-19.484
	p-value	0.000	0.299	0.001	0.829	0.036	0.001
D22	Coefficient	55.620	3.768	18.610	-1.023	-8.426	-19.054
	p-value	0.000	0.281	0.001	0.829	0.045	0.001
D23	Coefficient	57.430	3.968	20.379	-1.195	-8.334	-18.842
	p-value	0.000	0.280	0.001	0.810	0.056	0.001
D24	Coefficient	58.932	4.330	21.656	-1.040	-8.379	-18.752
	p-value	0.000	0.255	0.000	0.839	0.060	0.002
D25	Coefficient	59.962	4.531	22.623	-1.163	-8.229	-18.619
	p-value	0.000	0.248	0.000	0.826	0.070	0.002
D26	Coefficient	61.082	4.699	23.521	-1.108	-8.290	-18.527

	p-value	0.000	0.238	0.000	0.836	0.071	0.002
D27	Coefficient	61.724	4.778	24.156	-1.148	-8.161	-18.388
	p-value	0.000	0.238	0.000	0.833	0.079	0.002
D28	Coefficient	62.266	4.951	24.574	-1.108	-8.008	-18.351
	p-value	0.000	0.230	0.000	0.841	0.088	0.003
D29	Coefficient	63.154	4.891	24.862	-1.165	-8.213	-18.503
	p-value	0.000	0.236	0.000	0.833	0.082	0.003
D30	Coefficient	63.612	5.151	25.145	-0.998	-8.184	-18.491
	p-value	0.000	0.218	0.000	0.858	0.085	0.003
D33	Coefficient	84.892	-2.145	-1.995	-0.530	-0.977	0.716
	p-value	0.000	0.006	0.009	0.524	0.147	0.271
D36	Coefficient	85.440	-1.656	-4.868	-0.670	0.226	2.808
	p-value	0.000	0.003	0.000	0.247	0.592	0.000
D39	Coefficient	87.430	-1.452	-4.745	-0.750	0.108	2.831
	p-value	0.000	0.009	0.000	0.235	0.812	0.000
D42	Coefficient	89.100	-1.490	-4.698	-0.585	0.123	2.833
	p-value	0.000	0.013	0.000	0.389	0.807	0.001
D45	Coefficient	90.238	-1.522	-4.505	-0.805	-0.168	2.852
	p-value	0.000	0.003	0.000	0.137	0.658	0.000
D48	Coefficient	91.120	-1.733	-4.563	-0.598	-0.034	2.951
	p-value	0.000	0.001	0.000	0.210	0.919	0.000
D51	Coefficient	91.780	-1.534	-4.204	-0.563	-0.094	2.994
	p-value	0.000	0.001	0.000	0.222	0.776	0.000
D54	Coefficient	92.538	-1.481	-4.065	-0.825	-0.108	2.737
	p-value	0.000	0.001	0.000	0.070	0.724	0.000
D57	Coefficient	93.040	-1.600	-3.930	-0.693	-0.121	2.844
	p-value	0.000	0.000	0.000	0.082	0.655	0.000
D60	Coefficient	93.554	-1.413	-3.953	-0.815	-0.122	2.708
	p-value	0.000	0.001	0.000	0.058	0.667	0.000
