# Supplementary figures and images for: A Rational Approach to Predicting Immediate Release Formulation Behavior in Multiple Gastric Motility Patterns: A Combination of a Biorelevant Apparatus, Design of Experiments, and Machine Learning
Source: Pharmaceutics. 2023 Jul 31;15(8):2056. doi: 10.3390/pharmaceutics15082056 (PMC10458881; doi:10.3390/pharmaceutics15082056)

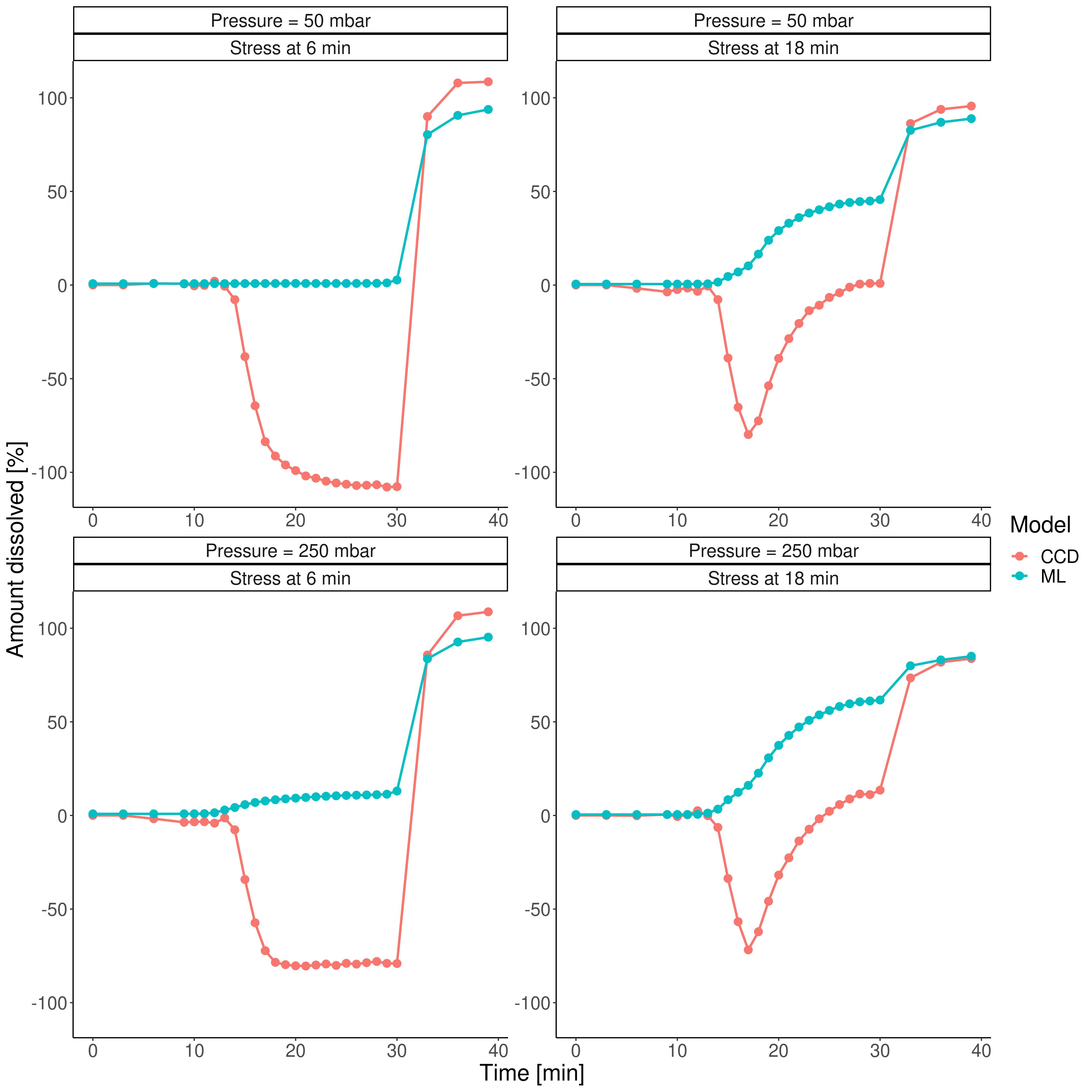

Supplement: Supplementary file 1 [file pharmaceutics-15-02056-s001.zip › Figrue S2_Out_of_range_predictions.jpg]

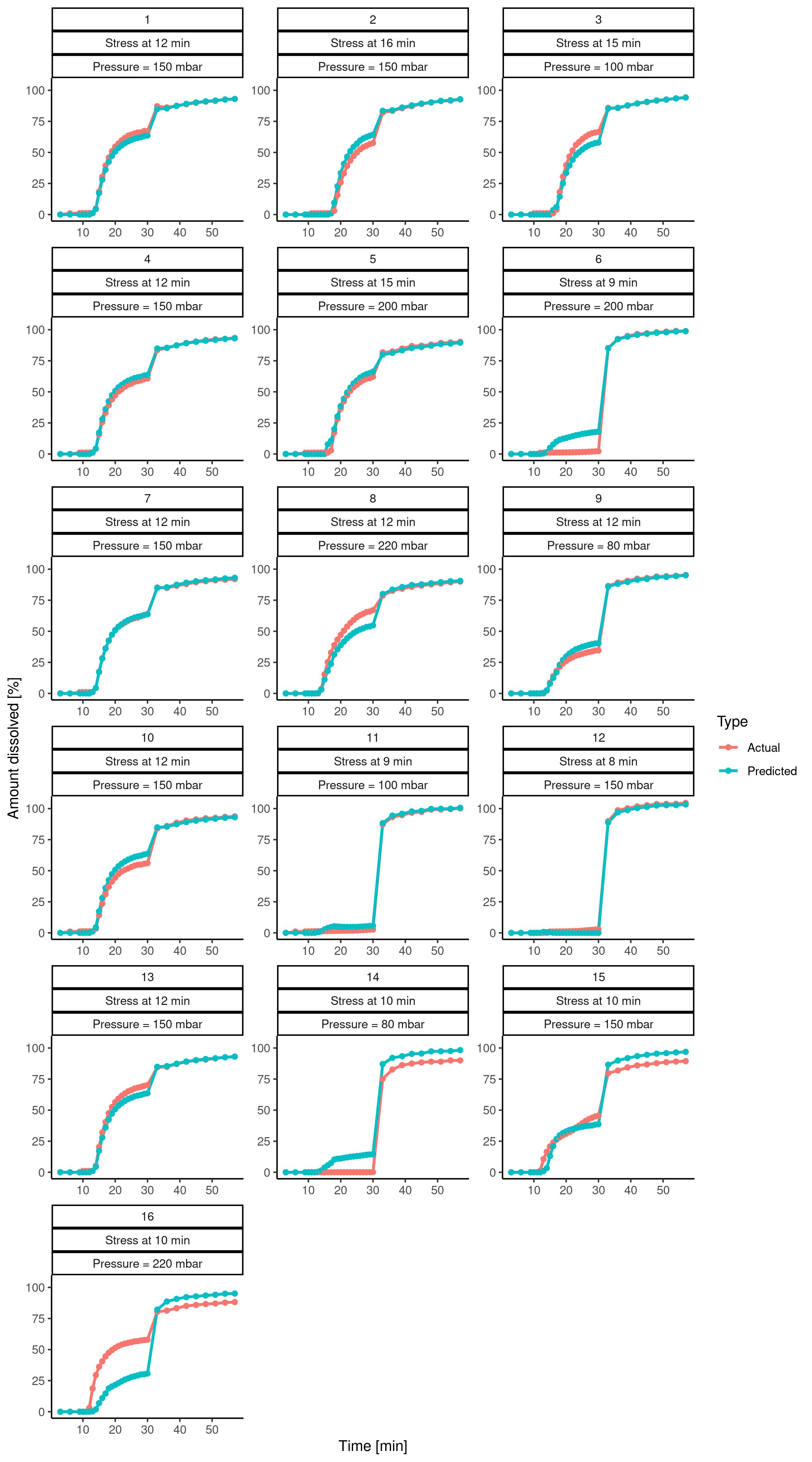

Supplement: Supplementary file 1 [file pharmaceutics-15-02056-s001.zip › Figrue S3_DoE_postdictions.jpg]

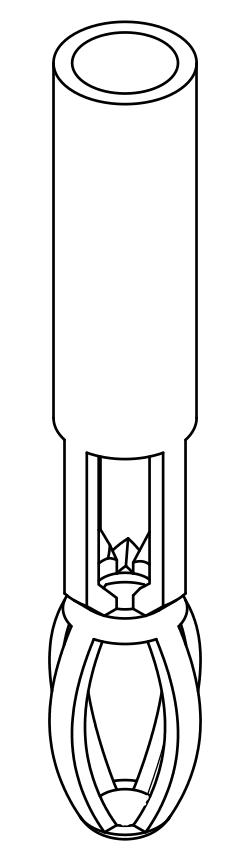

Supplement: Supplementary file 1 [file pharmaceutics-15-02056-s001.zip › Figure S1_Elastic_sinker.jpg]

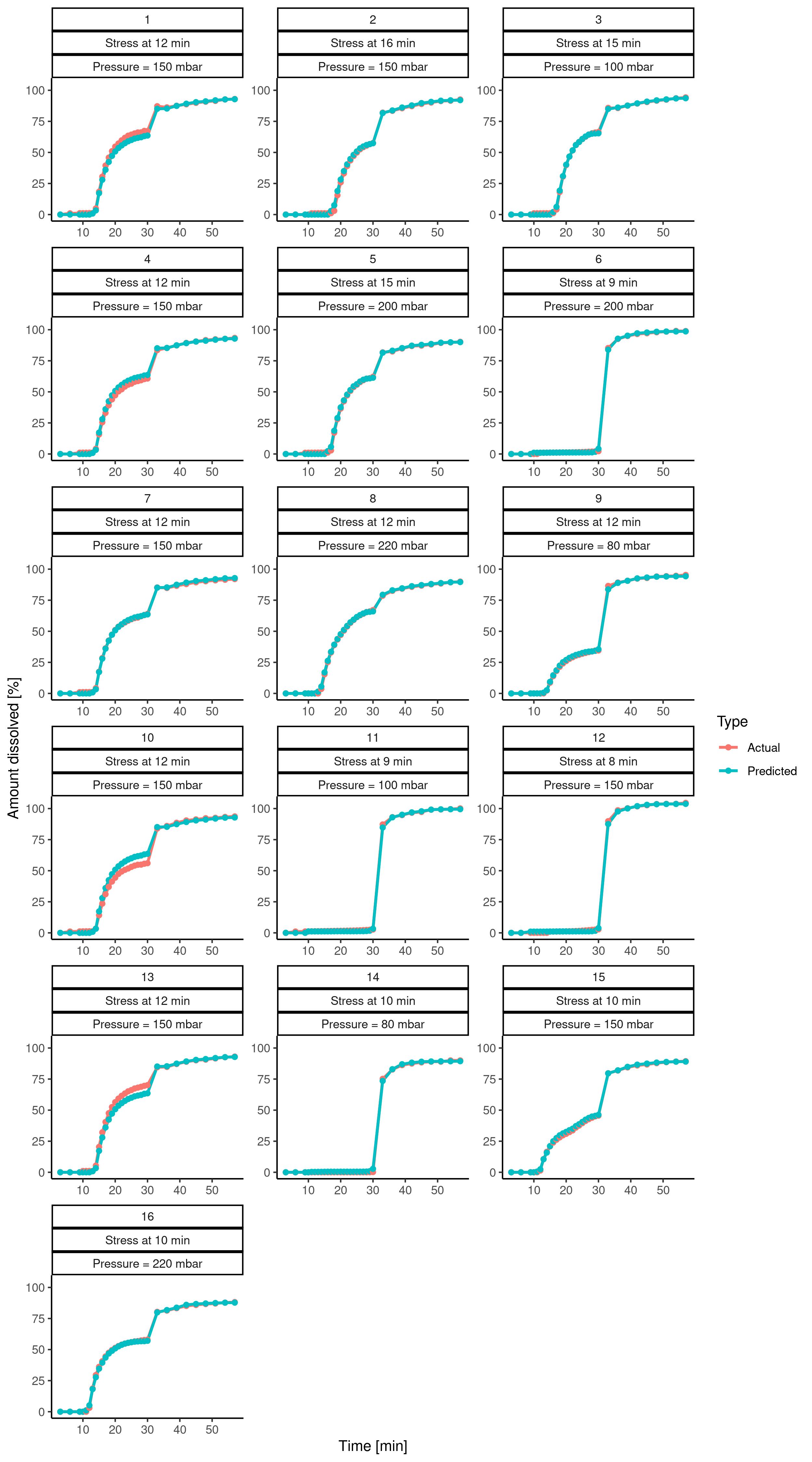

Supplement: Supplementary file 1 [file pharmaceutics-15-02056-s001.zip › Figure S4_ML_postdictions.jpg]
